# Ancient *Yersinia pestis* genomes lack the virulence-associated Ypf*Φ* prophage present in modern pandemic strains

**DOI:** 10.1098/rspb.2023.0622

**Published:** 2023-07-19

**Authors:** Joanna H. Bonczarowska, Julian Susat, Ben Krause-Kyora, Dorthe Dangvard Pedersen, Jesper Boldsen, Lars Agersnap Larsen, Lone Seeberg, Almut Nebel, Daniel Unterweger

**Affiliations:** ^1^ Institute of Clinical Molecular Biology, Kiel University, Rosalind-Franklin-Straße 12, Kiel 24105, Germany; ^2^ Unit of Anthropology, Department of Forensic Medicine, University of Southern Denmark, Odense M, 5230, Denmark; ^3^ Viborg Museum, Sct. Mogens Gade 5, Viborg 8800, Denmark; ^4^ Museum Horsens Arkæologisk Afdeling, Fussingsvej 8, Horsens 8700, Denmark; ^5^ Institute for Experimental Medicine, Kiel University, Michaelisstraße 5, Kiel 24105, Germany; ^6^ Max Planck Institute for Evolutionary Biology, August-Thienemann-Straße 2, Plön 24306, Germany

**Keywords:** *Yersinia pestis*, plague, pandemic, prophage, virulence factor, zonula occludens toxin

## Abstract

*Yersinia pestis* is the causative agent of at least three major plague pandemics (Justinianic, Medieval and Modern). Previous studies on ancient *Y. pestis* genomes revealed that several genomic alterations had occurred approximately 5000–3000 years ago and contributed to the remarkable virulence of this pathogen. How a subset of strains evolved to cause the Modern pandemic is less well-understood. Here, we examined the virulence-associated prophage (Ypf*Φ*), which had been postulated to be exclusively present in the genomes of strains associated with the Modern pandemic. The analysis of two new *Y. pestis* genomes from medieval/early modern Denmark confirmed that the phage is absent from the genome of strains dating to this time period. An extended comparative genome analysis of over 300 strains spanning more than 5000 years showed that the prophage is found in the genomes of modern strains only and suggests an integration into the genome during recent *Y. pestis* evolution. The phage-encoded Zot protein showed structural homology to a virulence factor of *Vibrio cholerae*. Similar to modern *Y. pestis*, we observed phages with a common origin to Ypf*Φ* in individual strains of other bacterial species. Our findings present an updated view on the prevalence of Ypf*Φ*, which might contribute to our understanding of the host spectrum, geographical spread and virulence of *Y. pestis* responsible for the Modern pandemic.

## 1. Introduction

*Yersinia pestis* is the pathogenic agent of plague—a zoonotic disease that can be transmitted from rodents to humans via a bite of an infected flea. This route of infection leads to bubonic plague. *Yersinia pestis* can also spread between humans resulting in the pneumonic plague. When the disease is untreated, the bacterium enters the bloodstream causing sepsis, which is also referred to as septicaemic plague. Although mortality rates of plague vary depending on the clinical form (30%–100%), *Y. pestis* infection is almost always fatal when no antibiotics are administered in a timely manner. The bacterium is responsible for at least three major pandemics in human history: the Justinianic plague (6th–8th c. AD), the Medieval plague (started in the 14th c. AD, subsequent sporadic outbreaks occurred until the 18th c. AD) and the Modern plague (end of 19th–mid-20th c. AD) [1–3]. Nowadays, *Y. pestis* still persists in environmental reservoirs around the globe [4] remaining a major threat to public health, as evidenced by the recent outbreaks of plague in Africa [5–7] and Asia [8].

*Y. pestis* evolved from *Yersinia pseudotuberculosis* approximately 7000 years ago [9] and gained high virulence by several genomic alterations. These changes include the inactivation of genes and the acquisition of additional virulence genes [10–12]. The impact of those genomic alterations on disease is most drastically demonstrated by their effect on the transmission routes of *Y. pestis* and the affected organs in humans. Acquiring *Yersinia* murine toxin (*ymt*), together with inactivating several other genes (*pde2*, *pde3*, *ureD*, *rcsA*, *flhD*), allowed efficient transmission of the pathogen via fleas from rodents to humans and resulted in infection of the lymphatic system [12,13]. The acquisition of the plasminogen activator *pla* enabled the bacterium to be transmitted by droplets from humans to humans resulting in airway infections and pneumonic plague [14,15]. Taken together, these findings describe the early stages of *Y. pestis* evolution – dating all the way back to the Neolithic period (approx. 3300–1200 BCE) [9,10,12,13,16]. As much as genetic alterations can aid the rise of pandemics, they might contribute to their fall and the extinction of strains. We recently reported the depletion of *pla* in medieval post-Black Death strains that have no modern descendants and seem to have gone extinct [17].

Unlike the genetic changes affecting *Y. pestis* virulence in its early phase of evolution [18], the more recent genome alterations that occurred between the Medieval and Modern pandemics are less well-understood. Among multiple diverse lineages of modern *Y. pestis,* only the strains of the 1.ORI phylogenetic group are thought to have caused the Modern plague pandemic [4]. Interestingly, the chromosome of 1.ORI strains encodes Ypf*Φ* or CUS-2 filamentous prophage [19–22]. By contrast to other types of bacteriophages, the filamentous phages do not kill the host and pose a relatively limited burden on the bacterium in a form of phage DNA replication and production of its proteins. Assembled virions are then egressed into the outside environment using pore-like channels (secretins) in the bacterium's wall [23]. In turn, the prophage-encoded genes can contribute to the host's virulence [19–21,24]. For instance, the CTX prophage of *Vibrio cholerae* encodes zonula occludens toxin (Zot) that increases permeability of epithelial barriers in the gut, leading to gastroenteritis [25]. Ypf*Φ* was also shown to enhance *Y. pestis* virulence, although the exact mechanism and its function remain unknown [21].

In this study, we reconstructed two new *Y. pestis* genomes from medieval/early modern Denmark, which were subjected to a comparative analysis together with over 300 previously published ancient, medieval and modern *Y. pestis* genomes. The examined bacterial genomes represent a timespan of over 5000 years. We focused on the virulence-associated filamentous phage (Ypf*Φ*) [19–21], which was absent from the genomes of all ancient and medieval strains.

## 2. Results

### (a) Ancient, medieval and early modern *Y. pestis* strains lack the virulence-associated prophage Ypf*Φ*

To characterize medieval and early modern *Y. pestis* genomes, we screened skeletal remains of 42 individuals (see Material) for molecular evidence of *Y. pestis* infection that were sampled from two medieval cemeteries in Denmark: Sct Trinitatis/Drotten in Viborg and Ødekirkegård in Sejet. Two individuals were excluded due to contamination, leaving 40 individuals in the analysis (17 females and 23 males) (electronic supplementary material, table S1). *Yersinia pestis* reads were noted in 25% (10/40) of individuals (4 females and 6 males) (electronic supplementary material, table S1). We successfully reconstructed two new pathogen genomes (table 1).

**Table 1.** Mapping statistics for two *Y. pestis* isolates from Denmark (Viborg and Sejet) with a mapping quality filter set to 30 (reference: CO92).

skeleton no. (Dating [cal AD])	site name (location no.)	genomic region	no. of aligned reads	mean coverage	coverage ≥1× [%]	coverage ≥2× [%]	coverage ≥3× [%]
**X52**	Sct. Trinitatis/Drotten, Viborg (VSM F902)	chromosome	516 891	7.0	94.8	93.1	89.6
[1432–1469]		pCD1	20 202	18.6	94.0	93.3	92.9
		pPCP1	107 07	72.7	80.9	80.8	80.8
		pMT1	17 892	11.6	94.0	94.0	93.3
**X3003**	Sejet Ødekirkegård (HOM 1046)	chromosome	1 215 553	17.42	95.2	94.9	94.7
[1490–1646]		pCD1	53 758	52.04	95.3	94.9	94.4
		pPCP1	14 843	103.82	81.3	80.9	80.9
		pMT1	24 813	16.79	94.2	94.1	94.1

**Figure 1.**
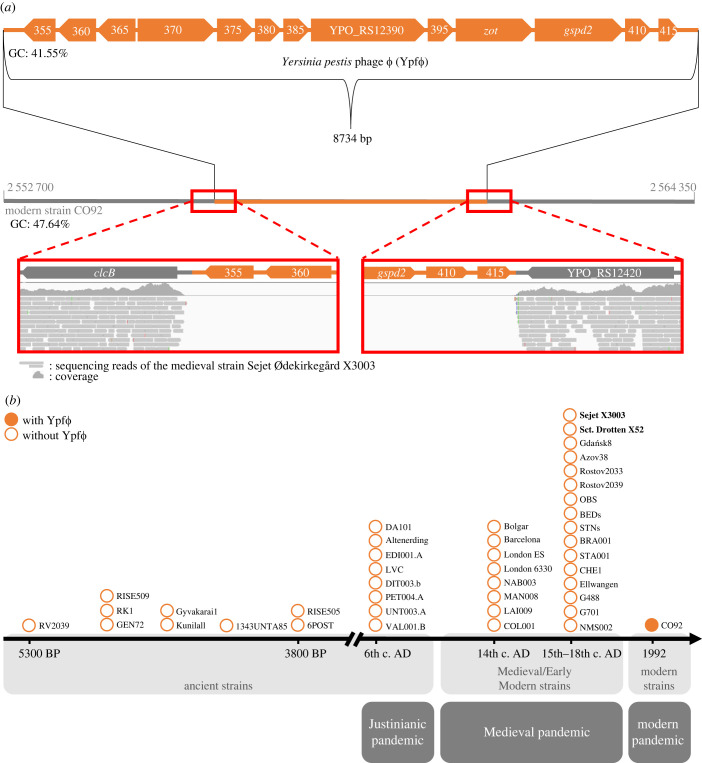
Ypf*Φ* of the modern strain CO92 is absent from ancient and medieval *Y. pestis* strains. (*a*) Ypf*Φ* of modern *Y. pestis* strain CO92. Sequencing reads of the medieval strain Sejet Ødekirkegård X3003 (as a representative strain) map to adjacent genes (coloured in grey) but not to the phage (coloured in orange) of the modern reference strain. Individual genes are labelled with their names or locus tags. The positions of the visualized genomic region are indicated. The G + C ratio was calculated using the online GC Content Calculator [26]. (*b*) Ancient and medieval strains lack Ypf*Φ* (empty circles). Individual strains are indicated along a timeline based on their estimated dating. The medieval Danish strains generated in this study (Sejet Ødekirkegård X3003 and Viborg Sct. Trinitatis/Drotten X52) are marked in bold. BP, before present; c. century; AD, anno domini.

To explore genomic differences between modern and medieval/early modern *Y. pestis*, the sequence alignments between the Danish isolates and the modern reference CO92 were inspected for gaps in coverage. An 8734bp-long genomic region was found in the modern CO92 chromosome that was not covered by any reads of the early strains (figure 1*a*). This region encompasses 13 genes (electronic supplementary material, table S2) and was previously identified as *Y. pestis* phage Ypf*Φ* or CUS-2 [19–22]. To test if the lack of Ypf*Φ* is specific to the Danish genomes or common among early modern, medieval and ancient *Y. pestis*, available sequencing reads of 45 medieval/early modern (6th–18th c. AD, electronic supplementary material, table S3) and 9 ancient (Neolithic and Bronze Age, electronic supplementary material, table S4) strains were additionally analysed. None of the ancient or medieval/early modern genomes carried Ypf*Φ* (figure 1*b*). This finding indicates that the absence of the phage seems to be a generalizable pattern among isolates spanning over five thousand years, including the first two plague pandemics.

### (b) A subset of modern *Y. pestis* strains stably integrated Ypf*Φ* in their genome

**Figure 2.**
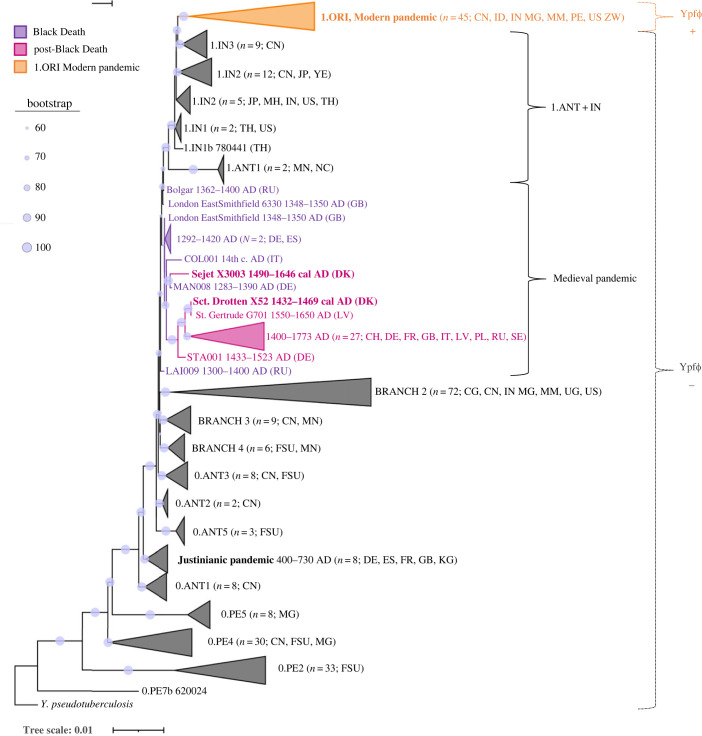
Maximum-likelihoodphylogeny of 255 modern and 47 medieval/early modern *Y. pestis* strains. The two Danish strains from Sejet Ødekirkegård and Viborg (Sct. Trinitatis/Drotten) are marked in bold. Medieval Black Death strains are shown in violet and the post-Black Death strains in purple. All strains carrying Ypf*Φ* in their genome belong to the modern phylogenetic group 1.ORI (orange). Remaining modern branch 1 strains form groups 1.ANT and 1.IN (1.ANT + IN). The tree is based on the SNP alignment of 10 315 positions with a bootstrap of 500 replicates and includes *Y. pseudotuberculosis* as an outgroup. The full tree with uncollapsed branches and bootstrap values can be found in the supplementary material (electronic supplementary material, figure S1).

To examine the prevalence of Ypf*Φ* among modern *Y. pestis* strains, 255 published genomes were screened for the phage (electronic supplementary material, table S3). The screening revealed Ypf*Φ* in the chromosomes of a subset of strains (*n* = 45; electronic supplementary material, table S5), which clustered together in one subbranch of branch 1 in the phylogenetic tree (figure 2; electronic supplementary material, figure S1). All isolates with Ypf*Φ* belonged to the phylogenetic group 1.ORI that is thought to be responsible for the Modern plague pandemic. All remaining branch 1 strains (1.ANT and 1.IN groups) did not carry the phage in their genomes and hereby will be referred to as the 1.ANT + IN strains. Although two previous studies suggested an extrachromosomal presence of Ypf*Φ* in several modern strains from different phylogenetic groups based on a PCR-analysis [21,27], our genome-based approach did not detect such extrachromosomal elements. This discrepancy could be explained by differences in the applied methods and the strains examined. Unfortunately, genome sequences are not available of the non-1.ORI strains that had been analysed by PCR.

Next, we analysed the intraspecific diversity of Ypf*Φ* among the different strains. Our results revealed the existence of multiple Ypf*Φ* variants in the existing assembled sequences (electronic supplementary material, figure S2, table S6). Some strains encoded two copies of the phage in a row (e.g. Java9) whereas other strains encoded one copy of the entire phage followed by an incomplete second copy (e.g. IP275). Moreover, pseudogenes in Ypf*Φ* of several strains were observed. In strains with two copies of Ypf*Φ*, one copy often contained a pseudogene whereas the other copy was marked as functional. This observation might indicate selection for one fully functional Ypf*Φ*.

In the phylogeny, the two new Danish genomes (Sejet Ødekirkegård X3003 and Sct. Trinitatis/Drotten X52, Viborg (VSM F902)) without Ypf*Φ* clustered within the known diversity of medieval *Y. pestis* strains (figure 2). Interestingly, although the C14 dating (electronic supplementary material, Excel sheet S1) places both strains in the post-Black Death period of the pandemic (1354 AD to 18th c. AD), Sejet X3003 clustered together with *Y. pestis* isolates responsible for the Black Death (1346–1353 AD). In addition, unlike Sct. Drotten X52, Sejet X3003 did not exhibit depletion of the *pla* region in the pPCP1 plasmid that was shown to be characteristic for the strains of the post-Black Death period [17]. These findings suggest that the Black Death *Y. pestis* lineage persisted in Denmark for at least over 150 years.

### (c) Common ancestry of Ypf*Φ* in *Y. pestis* and other *Enterobacteria*

**Figure 3.**
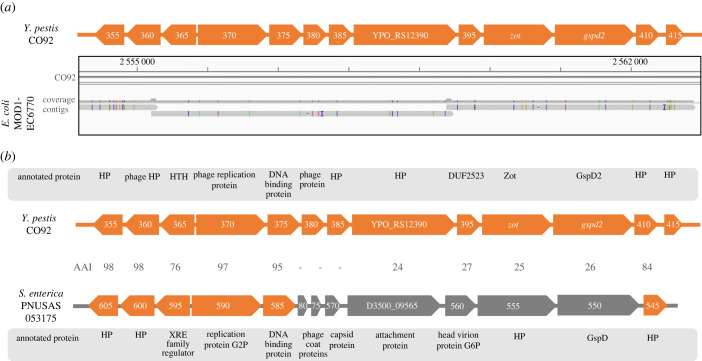
Ypf*Φ* is encoded by other *Enterobacteria*. (*a*) Alignment of Ypf*Φ* from *E.coli* MOD1-EC6770 (three contigs) and *Y. pestis* CO92 (NC_003143.1). SNPs are marked in colours along the contigs. A graphical depiction of the phage is shown above the alignment in orange. The numbers on the gene symbols have the prefix ‘YPO_RS12’ that was removed for clarity. In the NCBI the annotation for *zot* and *gspd2* are YPO_RS12400 and YPO_RS12405, respectively. (*b*) *S. enterica* PNUSAS053175 carries a putative phage with a similar genetic architecture to Ypf*Φ*. A graphical depiction of the respective genes and annotation is shown. The numbers on the gene symbols have the prefix ‘D3500_09’ that was removed for clarity. Genes in the phage of *S. enterica* that encode proteins exhibiting at least 30% amino acid identity in pairwise alignments with the respective proteins of *Y. pestis* are marked in orange. Amino acid sequence identities (AAI [%]) are indicated. HP stands for hypothetical protein.

To determine a possible origin of Ypf*Φ* in *Y. pestis*, the NCBI database was screened for bacterial strains carrying analogous sequences. We found a highly similar genomic region in the *Escherichia coli* strain MOD1-EC6770 (figure 3*a*). Unlike in previous observations [20], sequencing reads and contigs of the identified *E. coli* isolate aligned to full-length Ypf*Φ* of *Y. pestis* CO92 with high nucleotide identities (figure 3*a*, S3). This finding suggests a common ancestry of both phages in *E. coli* and *Y. pestis*.

Slightly truncated forms of Ypf*Φ* are also found in other *Enterobacteria* such as *Enterobacter ludwigii* and *hormarcheri*, *Morganella morganii*, *Klebsiella aerogenes*, *Salmonella enterica*, *Citrobacter amalonaticus, koseri* and *portucalensis* as well as *E. coli* and *Cedecea davisiae*. Those truncated forms encode 11 genes and lack equivalents to the two open reading frames at the 5′ end of *Y. pestis* Ypf*Φ*, namely YPO_RS12355 and YPO_RS12360. In *Citrobacter freundi, Enterobacter cloacae* and *Shigella sonnei* strains, we also found multiple copies of the phage. An alignment of the Ypf*Φ*s of various species reveals over 98.7% nucleotide identity over the 11 conserved genes (electronic supplementary material, table S7) and suggests high conservation across a wide range of enterobacterial isolates of mostly human origin.

The Ypf*Φ* of *Y. pestis* might belong to a large family of phages with a modular genomic architecture. The YpfΦ-like phage in *S. enterica* PNUSAS053175 encodes common and distinct features to Ypf*Φ* of *Y. pestis* (figure 3*b*). Whereas the first five genes (supposedly involved in phage regulation and replication) share high homology between the two phages, the proteins encoded in the remaining eight predicted open reading frames (mediating phage morphogenesis and secretion) differ strongly and share less than 30% amino acid identity. Interestingly, the *S. enterica* strain does not encode a homologue to *zot* and future work will need to resolve whether the proteins encoded by the YpfΦ-like phages also act as toxins. In the following, we examine the genes encoded by Ypf*Φ* to better understand its potential role in *Y. pestis*.

### (d) Ypf*φ* encodes proteins with structural homology to zonula occludens toxin and T2SS secretin

**Figure 4.**
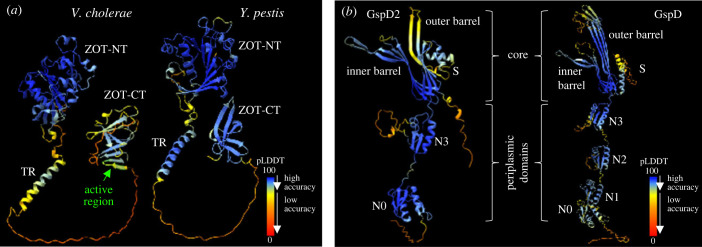
YpfΦ-encoded molecules exhibit homology to Zot—a virulence factor of *V. cholerae* (*a*) and secretin of the T2SS (*b*). Predicted structures are coloured based on the pLDDT confidence measure. (*a*) Predicted three-dimensional protein structure of *Y. pestis*'s Zot reveals a partial structural similarity to Zot of *V. cholerae*. Both proteins have a transmembrane region (TR) as well as C-terminal (ZOT-CT) and N-terminal (ZOT-NT) domains. The *Y. pestis’* Zot lacks the active region (FCIGRL) present in *V. cholerae*. (*b*) Predicted three-dimensional protein structures reveal differences in the protein core as well as in the periplasmic domain that is relevant for substrate specificity. The main domains (Nx and S) are labelled. The nomenclature is based on the resolved structure of the GspD of *Klebsiella pneumoniae* [31].

Interestingly, one of the Ypf*Φ* genes (*zot*) encodes a protein homologous to zonula occludens toxin (Zot) (electronic supplementary material, figure S4), which had recently been detected in the *Y. pestis* CO92 strain in an independent study [28]. 3D structure prediction of the *Y. pestis* and *V. cholerae* Zot proteins shows a partial structure similarity (figure 4*a*). Both proteins have the C-terminal (CT) and N-terminal (NT) domains that for *V. cholerae* were shown to reside in the bacterial periplasm and cytoplasm, respectively [29]. In *V. cholerae*, the CT domain contains the biologically active region (FCIGRL sequence at the 288–293 amino acid position), which modulates tight junctions of the epithelial cells [30]. Like other human pathogens, such as *Campylobacter concisus* and *Vibrio parahaemolyticus*, *Y. pestis*'s Zot lacks the FCIGRL (electronic supplementary material, figure S4). The presence of the FCIGRL sequence is, however, unnecessary for the Zot-mediated disruption of tight junctions for those bacterial species [31]. Thus, the activity of *Y. pestis*'s Zot might also be mediated via a different active site.

In addition to Zot, Ypf*Φ* encodes a gene for a protein that is annotated as a type II secretion system (T2SS) secretin and that we call GspD2 (figure 4*b*). The chromosome of *Y. pestis* already encodes a protein called GspD in a gene outside of Ypf*Φ* (electronic supplementary material, figure S5A). To assess the difference between the two proteins, their size was compared and an alignment of the amino acid sequences of GspD and GspD2 was performed (electronic supplementary material, figure S5B). While GspD is built of 640 amino acids (aa), GspD2 is smaller (414 aa). Only 26% amino acid identity was detected between the two proteins in the alignment.

To further test if YpfΦ-encoded GspD2 and GspD share similar three-dimensional structures despite sequence dissimilarities, the structures of the two *Y. pestis* proteins were modelled (figure 4*b*)*.* The three-dimensional models of the two proteins look surprisingly similar given the low amino acid sequence identity. Both proteins form a core with an inner and outer barrel as well as the N-terminal extensions of alpha helices. In comparison to GspD, GspD2 lacks two periplasmic domains (N1 and N2) and exhibits an outer barrel of reduced complexity. However, the pLDDT score, that is a measure of prediction accuracy, shows a relatively low confidence for the model of the GspD2 outer barrel. Apart from the GspD2 outer barrel, however, the pLDDT indicates a good prediction for both proteins. Both GspD and GspD2 consist of domains characteristic for an outer membrane secretin channel of T2SS, including the periplasmic domain and the domains forming the pore structure (the barrels and the S domain) [32]. In sum, the high similarity in overall structure, reflected in the presence of domains typical for T2SS secretin, suggests that GspD and GspD2 may both function as such, although with different substrate specificities as determined by the N-terminal domains in the periplasm. Based on a possible role of Zot in phage assembly and release [33] (in reference 33 Zot is annotated as YPO2279), GspD2 might form a complex with Zot to facilitate the secretion of Ypf*Φ*.

### (e) *Yersinia pestis* strains with and without Ypf*Φ* differ in their host spectrum

**Figure 5.**
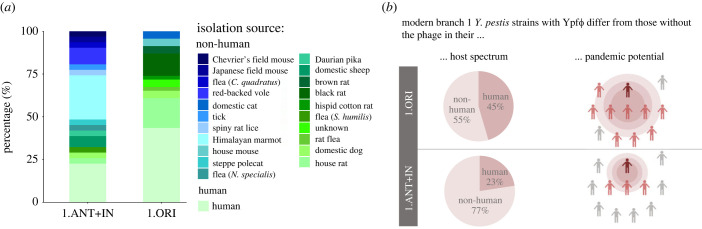
Host spectrum of YpfΦ-negative and YpfΦ-positive strains of branch 1. (*a*) Proportion of different hosts of 1.ANT + IN (YpfΦ-negative, *n* = 31) and 1.ORI (YpfΦ-positive, *n* = 45) strains analysed in this study. Isolation sources are indicated with respective colours. More details about the sources can be found in electronic supplementary material, table S8. (*b*) Differences between the YpfΦ-positive and YpfΦ-negative *Y. pestis* strains with regards to the host spectrum and pandemic potential. The number of different hosts for 1.ORI was 9, while for 1.ANT + IN it was 15. Illustrations were created with BioRender.com.

To better understand the potential impact of the prophage Ypf*Φ* on bacteria beyond its known association with virulence [21], we determined features that are associated with modern YpfΦ-positive strains (1.ORI) and are absent from modern YpfΦ-negative branch 1 strains (1.ANT + IN). Of particular interest is the source of the analysed isolates, as *Y. pestis* is found in multiple animal hosts besides humans. These animal hosts serve as natural reservoirs of this pathogenic bacterium and play a key role during transmission. When comparing the host spectrum between strains with and without the phage, we found YpfΦ-positive strains (1.ORI) predominantly among animals associated with human habitats (no. of hosts = 9), like rats, mice, cats and dogs, whereas YpfΦ-negative isolates are found among more diverse animals (no. of hosts = 15) (figure 5*a*; electronic supplementary material, table S8). Furthermore, the proportion of the human host was higher for the YpfΦ-positive strains (20/44, 45.45%) relative to the YpfΦ-negative strains (7/31, 22.6%) (figure 5*a*). Although a strong trend was noted, it was not statistically significant (*p* = 0.0527). However, the proportion of human isolates among YpfΦ-positive bacteria that is seen here is likely an underestimate, considering that the analysed isolates were chosen to represent maximal host diversity. The influence of sampling bias cannot be excluded, as in many cases the isolate source is unknown or the host's natural habitat is undefined, i.e. the animal can be found in both human-associated and wild environment (electronic supplementary material, table S8). Nevertheless, we observe an altered host range among isolates with and without the phage in the analysed sample (figure 5*b*). Another feature of the YpfΦ-positive strains is that they all belong to the phylogenetic group 1.ORI which has been linked with the Modern plague pandemic and subsequent disease outbreaks (figure 5*b*).

## 3. Discussion

In this study, we confirm that Ypf*Φ* is associated with *Y. pestis* responsible for the Modern plague pandemic (1.ORI) (figure 2). Furthermore, the presence of Ypf*Φ* in modern *Y. pestis* strains of the 1.ORI group correlates with their seemingly altered host spectrum and pandemic potential relative to other branch 1 modern *Y. pestis* strains (YpfΦ-negative) (figure 5). Ypf*Φ* was also absent in the genomes of strains which were responsible for the Medieval and Justinianic plague pandemics as well as those in the Neolithic or Bronze Age (figure 1*b*). The lack of coverage of Ypf*Φ* genes in medieval strains can also be seen in the data published by Seguin-Orlando and colleagues (fig. 4A in Seguin-Orlando *et al*., [34]). As the phage is only present in one modern phylogenetic sub-branch, it is possible to estimate the approximate time of its incorporation based on the molecular dating of the splits in the phylogeny. Molecular dating depends on various factors, such as the included strains and the methods used for the analysis, and thus there seems to be no consensus in the literature concerning the exact date of the 1.ORI branch split [35]. Although the 1.ORI branch could have emerged anytime between approximately 550 and 150 years ago [9,35–37], it is likely that both the split and the genomic acquisition of Ypf*Φ* occurred before the Modern plague pandemic.

As previous functional studies in mice showed that Ypf*Φ* enables better colonization of the host and confers increased fitness during infection of mammals [21], the chromosomal acquisition of the phage likely influenced the pathogenicity of the bacterium towards humans. Changes in pathogenicity could possibly affect the clinical manifestation of plague, suggesting possible differences between the ancient (Justinianic and Medieval) and Modern pandemics. Differences in pathogenicity do not necessarily have to be reflected in the estimated mortality rates of the pandemics, as various factors, such as proximity to zoonotic reservoirs, climate, settlement type/crowding, health status and behaviour-related exposure, can influence the outbreak outcomes (e.g. [38–42]). This finding highlights the introduction of potential bias when interpreting various historical sources on plague epidemiology in the past based on the inference from knowledge about modern plague.

*Y. pestis* has evolved its remarkable virulence and transmission abilities via horizontal acquisition of large pieces of foreign DNA, such as plasmids (pPCP1, pPMT1) and pathogenicity islands. These alien genetic elements, while non-essential for survival, were probably crucial for the emergence of pathogenic *Yersinia* in general [43,44]. For instance, the virulence of pathogenic *Yersinia* (*Y. pestis, Y. pseudotuberculosis* and *Y. enterocolitica*) strictly depends on the presence of the pCD1 plasmid [44]. Furthermore, within these three species, certain subgroups have acquired a genomic region which allows a systemic dissemination of the bacterium and confers the high-virulence phenotype. Therefore, the region is called a high pathogenicity island (HPI) [45]. Both HPI and, to a lesser extent, Ypf*Φ* are linked to an increased fitness during the infection process and their genomic acquisition represents rapid modification in bacterial pathogenicity [21,44–46]. By contrast to the relatively well described role of the HPI and the *Y. pestis* plasmids in the bacterium's virulence and transmission, the mechanisms responsible for the increased dissemination of the pathogen that is conferred by Ypf*Φ* have so far remained unknown. Here, we show that Ypf*Φ* encodes a protein homologous to zonula occludens toxin (Zot) that is a virulence factor in other pathogenic bacteria, such as *Neisseria meningitidis*, *Acinetobacter baumanii*, *Salmonella enterica*, *Burkholderia cenocepacia* and *Vibrio cholerae*, in which it had first been identified [25,28]. In these pathogens, Zot increases permeability of the gut epithelia [25], the mucosa [47] and endothelial cells in the brain [48]. Based on our findings, we propose that proteins encoded in the phage (i.e. Zot and GspD2) represent good candidates for further experimental studies on the molecular mechanism of phage-mediated virulence.

Similar to HPI [44,46], Ypf*Φ* is found across various bacterial genera (electronic supplementary material, table S6) [20,28]. Presence of complete Ypf*Φ*, as seen in *Y. pestis*, in the genome of *E. coli* MOD1-EC6770 suggests this species as a possible source of the Ypf*Φ* in *Y. pestis*. Such horizontal spread of large genetic elements, like prophages, plasmids and pathogenicity islands, often cause ‘quantum leaps’ in evolution [49], affecting the bacterium's metabolism, transmission and virulence. For *Y. pestis*, the acquisition of pPCP1 and pMT1 plasmids as well as HPI allowed for a flea- and airborne transmission and rapid systemic dissemination of the pathogen—a deadly combination that kills up to 100% of infected individuals [50]. In recent years, several *Y. pestis* strains acquired additional plasmids (pIP1202, pIP203 and pIP2180H) that confer the ability to resist antibiotic treatment [51]. Ypf*Φ* likely represents another ‘leap’ in the evolution of the pathogen. A better knowledge on the recent evolution of *Y. pestis* is key to the understanding of the bacterium's pathogenicity. Acquisition of foreign genetic material, which can include virulence factors, might lead to unusual clinical forms of plague that can be challenging to diagnose. For instance, during a plague-related outbreak of gastroenteritis in Afghanistan, 20.5% of the infected individuals (17/83) died before the appropriate treatment was applied [52]. If the YpfΦ-encoded Zot indeed disrupts the tight junctions of epithelial cells, like in several other bacteria [25,31], the gastroenteritis can be a result of an infection with the phage-positive *Y. pestis*.

## 4. Material

The skeletal material used for *Y. pestis* screening comprised 74 bones and teeth belonging to 42 individuals from two medieval/early modern parish cemeteries (1000–1575 AD) [53–56] in Denmark (electronic supplementary material, table S1). The skeletal material analysed in this study is stored at the ADBOU skeletal collection (Department of Forensic Medicine, University of Southern Denmark).

The Ypf*Φ* distribution across the *Y. pestis* strains was analysed among previously published 255 modern, 45 medieval/early modern (6th–18th century AD; electronic supplementary material, table S3) and 9 ancient (Neolithic and Bronze Age, electronic supplementary material, table S4) strains. Two new genomes (Sejet Ødekirkegård X3003 and Sct. Trinitatis/Drotten X52, Viborg (VSM F902)) were also analysed. Sequencing data was downloaded from online repositories such as the European Nucleotide Archive (ENA) and the National Center for Biotechnology Information (NCBI).

## 5. Methods

### (a) Processing of the metagenomic medieval/early modern samples

All DNA samples were extracted and processed in a dedicated ancient DNA facility at the University of Kiel following the guidelines on contamination control in ancient DNA [57–59], according to a previously published protocol for the non-UGD treated samples [60]. Shotgun sequencing was performed on the Illumina HiSeq 6000 (2 × 100) platform of the Institute of Clinical Molecular Biology in Kiel.

Adapter sequences were removed and paired-end reads were merged with ClipAndMerge v1.7.7. [61]. Shotgun sequence data was mapped to the human genome (build hg19) using BWA v0.7.12 [62] with a reduced mapping stringency parameter ‘-n 0.01’ to account for mismatches in aDNA. Duplicated reads were removed with DeDup v0.12.2 [61].

To confirm the ancient origin of the sequences, terminal damage of the reads (C to T substitutions) was assessed with DamageProfiler [63]. After the validation, the first two positions from the 5′ and 3′-ends of the reads were trimmed. Furthermore, X-chromosome and mitochondrial DNA contamination were assessed with ANGSD and Schmutzi, respectively [64,65].

### (b) Identification of *Y. pestis*-positive samples in medieval and early modern individuals

Initial screening of all samples for the presence of *Y. pestis* DNA was performed with Megan Alignment Tool 0.3.0 (MALT) [66] (SemiGlobal alignment mode, identity threshold = 90%), using a custom database containing bacterial genomes available at the NCBI platform (24.01.2019). Output alignments were inspected visually in MEGAN 6 [67]. The samples that contained reads aligning to *Y. pestis* were further evaluated.

The *Y. pestis*-positive status was based on detection of reads unique for the pathogen in the sample. *Y. pestis*-specific reads were obtained in competitive mapping against *Y. pestis* (NC_003143.1, NC_003131.1, NC_003134.1, NC_003132.1) and *Yersinia pseudotuberculosis* (NC_006155.1) reference genomes, using Burrows-Wheeler Aligner (BWA) v0.7.12 (*n* = 0.01, l = 300) [62]. Output BAM files were then filtered for quality 30 with SAMtools [68]. Subsequently, the number of *Y. pestis-*specific reads was noted with *samtools idxstats*.

### (c) Alignment and detection of Ypf*Φ*

Sequencing data from all *Y. pestis* strains (electronic supplementary material, table S3 and S4) were mapped against the CO92 reference genome (NC_003143.1, NC003131.1, NC_003132.1, NC_003134.1) with BWA v0.7.12 [62]. For ancient data, a reduced stringency parameter was used (-n 0.01) to account for mismatches in ancient DNA and two positions from the 5′ and 3′-ends of the reads were trimmed. Duplicated reads were removed with DeDup v.0.12.2 [61]. Regions with zero coverage were identified and the samples with no reads mapping to the NC_003143.1 : 2554178–2562912 region were classified as strains without Ypf*Φ*. Moreover, sequencing data of an example strain CMCC10012 (not carrying the phage) was mapped in a competitive mapping against the CO92 reference genome and the CO92 chromosome without the 2554178–2562912 region. This way, unique gap-bridging reads were identified for the CMCC10012, confirming the lack of Ypf*Φ*.

### (d) Analysis of Ypf*Φ*

Reads mapping to the CO92 Ypf*Φ* region were extracted from a randomly chosen example strain (EV76). The phage reads were then mapped against previously published genome assemblies and contigs of the YpfΦ-positive *Y. pestis* strains (electronic supplementary material, table S5) with BWA v0.7.12 (*n* = 0.01, l = 300) [62] to locate the position of Ypf*Φ* within the chromosome of each strain. Knowing the phage locus for the strains, genome annotation was inspected in the graphic view panel of the sequences in NCBI to identify the Ypf*Φ* variants. Association with a particular phylogenetic group was made based on available data [37] or the position in the phylogeny. To identify similar sequences in other bacterial species, the extracted phage reads were blasted with the BLASTn online tool (default parameters) against the nucleotide collection (nt).

Differences in host diversity between modern 1.ORI and 1.ANT + IN strains were assessed with Fisher's exact test using IBM SPSS Statistics (v. 26).

### (e) Comparative analysis of Zot and secretins

Three-dimensional (3D) structures of CO92 *Y. pestis* secretins (GspD and GspD2) as well as *Y. pestis* and *V. cholerae* Zot molecules were predicted using Alpha Fold with default parameters [69]. MUSCLE online tool [70] (ClustalW) was used for multiple sequence alignment of amino acid sequences.

### (f) Phylogenetic analysis

Phylogenetic analysis was performed with RAxML [71] using the GTRGAMMA model with 500 bootstrap replicates. MultiVCFAnalyzer [72] was used to generate a SNP-based multiple alignment of 255 previously published modern and 47 medieval/early modern *Y. pestis* strains (including the two Danish genomes reconstructed in this study: Sejet Ødekirkegård X3003 and Sct. Trinitatis/Drotten X52, Viborg (VSM F902) with *Y. pseudotuberculosis* (NZ_CP008943.1) as an outgroup (electronic supplementary material, table S3). The input VCF files were generated with the UnifiedGenotyper module from the Genome Analysis Toolkit (GATK) v3.6 [73]. A SNP was called if the position was covered by at least three reads, the genotype quality was at least 30 and the fraction of mapped reads containing the SNP was at least 90%.

## Data Availability

Sequences used in the reconstruction and subsequent analysis of *Y. pestis* genomes from Sejet X3003 and Viborg X52 are available through the European Nucleotide Archive (http://www.ebi.ac.uk/ena/browser/view/) under Accession Number PRJEB60595. The data are provided in electronic supplementary material [74].
